# High-fat diet–induced vagal afferent dysfunction via upregulation of 2-pore domain potassium TRESK channel

**DOI:** 10.1172/jci.insight.169516

**Published:** 2023-02-22

**Authors:** Gintautas Grabauskas, Xiaoyin Wu, ShiYi Zhou, JiYao Li, Jun Gao, Chung Owyang

Original citation: *JCI Insight*. 2019;4(17):e130402. https://doi.org/10.1172/jci.insight.130402

Citation for this retraction: *JCI Insight*. 2023;8(4):e169516. https://doi.org/10.1172/jci.insight.169516

The University of Michigan recently notified *JCI Insight* of concerns regarding the identity of the antibodies used in Figure 2. *JCI Insight* previously issued a corrigendum to update the source of the TRESK and CCK-AR antibodies used for immunocytochemistry; however, the antibodies listed in the corrigendum were not correct (1). The institutional investigation determined that there was falsification of immunocytochemistry data by the improper description of the species of antibody used in Figure 2 and that falsified data were intentionally, knowingly, or recklessly included in the publication. Due to the intentional inclusion of falsified data and continued concerns over the identity of antibodies, *JCI Insight* is retracting this article.

## Version 1 02/22/2023

Electronic publication

